# Traffic accidents under the influence of alcohol

**DOI:** 10.17886/RKI-GBE-2016-027

**Published:** 2016-09-28

**Authors:** Anke-Christine Saß, Martina Rabenberg, Alexander Rommel

**Affiliations:** Robert Koch Institute, Department for Epidemiology and Health Monitoring, Berlin, Germany

**Keywords:** TRAFFIC ACCIDENTS, ALCOHOL, ADULTS, MEN, GERMANY

## Abstract

In 2014, 260 persons were killed in Germany in road traffic accidents under the influence of alcohol. Compared to road traffic accidents in general, accidents under the influence of alcohol frequently have particularly serious consequences. In 2014, police established that 13,742 persons were involved in traffic accidents while under the influence of alcohol. Just under 40% of these were young men aged 18 to 34 years. The number of road traffic accidents under the influence of alcohol has been declining for over 20 years now and in 2014 reached its lowest level since the recording of the relevant data began.

## Introduction

High-risk, abusive and addictive consumption of alcoholic beverages entails huge risks to the health of consumers, may be harmful to third parties, has an impact on the social environment of the consumers and causes substantial costs on a macroeconomic scale [1]. Therefore, the prevention of alcohol-related consequences represents a key element of numerous public health strategies. The “Global Action Plan for the Prevention and Control of Non-Communicable Diseases 2013–2020” launched by the WHO calls for a relative reduction in high-risk alcohol consumption by 10% from the year 2010 to 2025 [2]. In Germany, the need to combat alcohol consumption and its consequences has been incorporated in the national health targets [3].

In Germany, a total of 260 persons were killed in road traffic accidents under the influence of alcohol in 2014. Alcohol-related accidents constitute a risk to life and health and are avoidable. In Germany, according to Section 24a of the Road Traffic Act it is an offence for drivers to operate a motor vehicle with 0.25 mg/l or more of alcohol on their breath or a level of 0.5 parts per thousand of alcohol in their blood. Road users involved in a traffic accident are considered to be under the influence of alcohol even at lower alcohol values. A zero alcohol limit has been stipulated for learner drivers. Some European countries have lower alcohol limits and harsher penalties than Germany [4]. Controls and fines are key measures to prevent driving under the influence of alcohol and to reduce the number of alcohol-related traffic accidents. Furthermore, there are additional measures, such as those within the scope of the federal German Government’s road traffic safety work which it conducts jointly with numerous organisations in society. In its “Road Traffic Accident Prevention Report” (Unfallverhütungsbericht Straßenverkehr), the Federal Government provides information each year on measures in the field of accident prevention [5]. The focal points of work in the field of alcohol prevention vary from time to time. In the current reporting period 2012/13, issues included the introduction of alcohol ignition locks (or alcohol interlocks) for rehabilitation of drivers who have come to the attention of the police, and these locks have proved to be helpful as part of the project. In addition, the Federal Government is involved in the EU project DRUID, which develops recommendations for identifying, punishing and rehabilitating drivers under the influence of psychoactive substances [5]. The Federal Centre for Health Information (Bundeszentrale für gesundheitliche Aufklärung) uses its campaign “Alcohol? Know your Limit” to address youths and young adults. Alcohol in road traffic is one of the topics dealt with on posters, on the campaign website and in video sequences [6]. The prevention programme P.A.R.T.Y. (“Prevent Alkohol and Risk Related Trauma in Youth”), calling for adolescents to spend a day in an accident clinic, the Internet database “Alcohol and Drugs in Road Traffic” (Alkohol und Drogen im Straßenverkehr) as well as various events and brochures for adults are coordinated and published by the German Traffic Safety Council (Deutscher Verkehrssicherheitsrat) [7].

This fact sheet provides a current overview of road traffic accidents under the influence of alcohol. The key contribution in the current edition of the Journal of Health Monitoring “High-risk alcohol consumption by adults in Germany; prevalences and long-term trends“ examines information on alcohol consumption in Germany. Moreover, this edition contains fact sheets on hospitalisations due to acute alcohol intoxication and on alcohol-related mortality.

## Indicator

The analysis is based on data of the 2014 Road Traffic Accident Statistics of the Federal Statistical Office. Amongst the information contained in these statistics are details of accidents involving personal injury on German roads, in the course of which at least one party to the accident was under the influence of alcohol. Accidents where the police were not notified are not included in the statistics. “Parties” are all drivers of motor vehicles or pedestrians who sustained or caused injuries themselves or whose vehicles were damaged. Below is a presentation of how many persons were involved in alcohol-related road traffic accidents in 2014 and how the case numbers are distributed by gender and age. In supplementation, selected additional key features of alcohol-related accidents are described, including information on long-term developments.

## Classification of findings

In 2014 there were 13,612 accidents involving personal injury in which at least one party to the accident was under the influence of alcohol [8]. In relation to the accident events, this means that alcohol was a cause in 4.5% of all cases involving personal injury. Of all terminal injuries 7.7 per cent were due to an accident caused to at least some extent by alcohol. The differing proportions are an indication of the above-average level of severity of accidents under the influence of alcohol.

Regionally there are substantial differences in the frequency of alcohol-related accidents: the lowest proportions of alcohol-related accidents involving personal injury were registered in Hamburg (3.1%) and Berlin (3.3%). The values recorded in the Saarland region (6.4%) and in Mecklenburg-West Pomerania (6.2%) were significantly higher than the federal average.

**Fig. 1:**
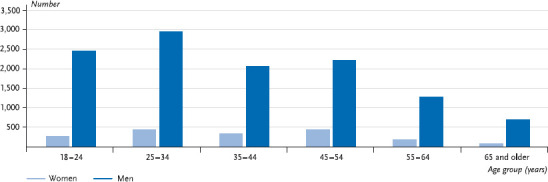
Persons involved in alcohol-related accidents by age group and gender Data basis: Road Traffic Accident Statistics Source: [8]

In focusing on prevention, evaluations regarding the persons involved in accidents are important. Which groups of persons were involved in accidents while under the influence of alcohol? In 2014, 13,742 persons under the influence of alcohol were involved in the accidents referred to above, of whom 86.8% were male. In particular, young men happen to be involved in traffic accidents involving drunkenness. Of all persons involved in accidents 40.2% were young men aged 18 to 34 years (Fig. 1). The number of drunken persons involved in accidents declines substantially for both genders from the age of 55.

An increased likelihood of men, especially young men, involved in accidents is also reflected in other results of the traffic accident statistics, e.g. in terms of the number of deaths. In 2014, 34 young women per million inhabitants in their age group were killed; for men, this value was more than three times as high (123 men aged 18 to 24 years) [9]. This is confirmed in surveys including other areas of accident events: according to a current survey representative of the population as a whole regarding the accident events (all accident locations), 10.9% of all men and 6.6% of women sustained an accidental injury that had to be treated by medical practitioners at least once a year. Of young men aged 18 to 29 years, as many as 20% had an accident (19.4%) [10]. The higher risk of accidents in men is explained by riskier behaviour patterns, amongst other factors. This “risk-seeking” behaviour that is exhibited in particular by younger men is considered a key factor for explaining the pronounced gender-specific frequencies of accidents [11]. Differences in drinking patterns and in risk for men and women were also observed with regard to alcohol consumption [12]. This is reflected in all surveys carried out in this regard. In the German Health Interview and Examination Survey for Adults (DEGS1), it was established that 13.1% of women and 18.5% of men aged 18 to 79 years consume over 10g (women) and 20g (men) of pure alcohol. Accordingly, this reflects a tendency towards risky consumption that is significantly more frequent amongst men than women. Binge drinking also occurs considerably more frequently in men than in women [13, 14] (see also focus in this issue). Evidently this is where areas of masculine risk behaviour accumulate.

In addition to personal characteristics such as age and gender, other factors in connection with alcohol-related accidents also provide indications where prevention must begin. Passenger car drivers (56.6%), both male and female, account for the largest proportion of parties to alcohol accidents involving personal injury, while more than a quarter are cyclists (25.7%), again both male and female. Drivers of road haulage vehicles account for a very small proportion of alcohol accidents (2.5%). They are subject to absolute prohibition of alcohol when exercising their activities; they are at risk of frequent inspections [8]. Time also plays a part as far as accident risks are concerned: alcohol-induced accidents occur especially often on weekends. Saturdays and Sundays are the days of the week that are most prone to accidents, with a share of 23.9% and 22.6%, respectively. Evaluations of the times of day show that alcohol-related accidents chiefly occur in the evenings and nights; this means that of all night-time accidents that occur between 22:00h and 06:00h, alcohol played a part in every fourth case (24.4%) [8].

A positive trend is discernible regarding the long-term development of traffic accidents under the influence of alcohol (Figs. 2a, b). The number has been declining for over 20 years now; in 2014 it reached the lowest level since the recording of the data began. This also applies to traffic accidents in general: the number of traffic casualties in 2014 was at the lowest point since the introduction of the official statistics. At present, accidents merit attention where these occur under the influence of intoxicating substances such as drugs and narcotics. In this respect an increase has been observed since the early 1990s, even though the case numbers are low. It is assumed that the estimated number of unreported or undetected cases is high in this field, as is the case with alcohol-related accidents [8]. Despite declining accident numbers, the improvement in traffic safety remains a task for society as a whole. The German Traffic Safety Council (Deutscher Verkehrssicherheitsrat – DVR) endorses the “Vision Zero” strategy: safe mobility in Germany, with no deaths and serious injuries in road traffic [15]. It also endorses a general prohibition of alcohol for drivers [16].

## Key statements

- In 2014, 260 persons were killed in Germany in road traffic accidents under the influence of alcohol.
- In 2014, police established that 13,742 persons were involved in traffic accidents while under the influence of alcohol. Just under 40% of these were young men aged 18 to 34 years.
- The number of road traffic accidents under the influence of alcohol has been declining for over 20 years now and in 2014 reached its lowest level since the recording of the relevant data began.

## Figures and Tables

**Fig. 2a:**
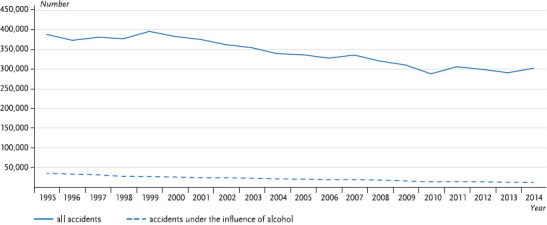
Number of road traffic accidents in the past 20 years (1995–2014) Data basis: Road Traffic Accident Statistics Source: [17]

**Fig. 2b:**
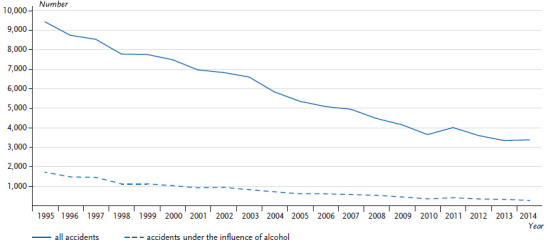
Number of persons killed in road traffic accidents in the past 20 years (1995–2014) Data basis: Road Traffic Accident Statistics Source: [17]
